# Supplementary material for: Thermal-healing of lattice defects for high-energy single-crystalline battery cathodes
Source: Nat Commun. 2022 Feb 4;13:704. doi: 10.1038/s41467-022-28325-5 (PMC8817033; doi:10.1038/s41467-022-28325-5)
Supplement: Supplementary file 1 — Supplementary Information [file 41467_2022_28325_MOESM1_ESM.pdf]

Supplementary Information for

Thermal-healing of lattice defects for high-energy single-crystalline battery cathodes

Shaofeng Li†, Guannan Qian†, Xiaomei He†, Xiaojing Huang, Zhisen Jiang, Yang Yang, Wei-Na Wang, Dechao Meng, Chang Yu, Sang-Jun Lee, Jun-Sik Lee, Yong S. Chu, Zifeng Ma, Piero Pianetta, Jieshan Qiu*, Linsen Li*, Kejie Zhao*, Yijin Liu*

†These authors contributed equally to this work.

*Correspondence and requests for materials should be addressed to J. Qiu (jqiu@dlut.edu.cn), L. Li (linsenli@sjtu.edu.cn), K. Zhao (kjzhao@purdue.edu), or Y. Liu (liuyijin@slac.stanford.edu)

Supplementary Figures

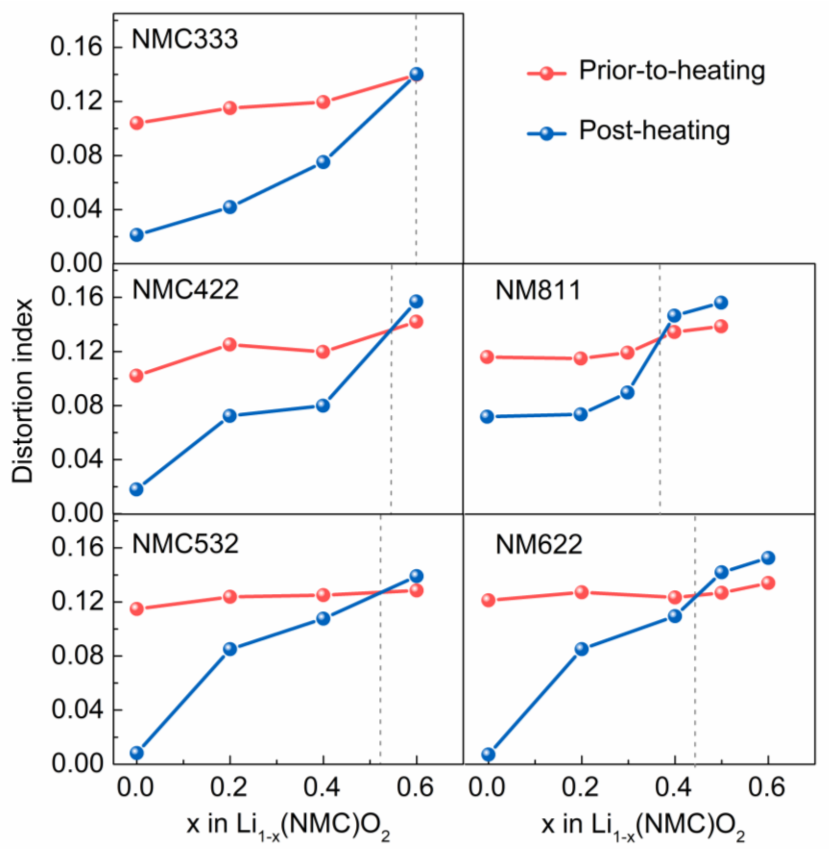

Supplementary Figure 1. Average distortion index d_{ave} of both prior-to-heating (red points) and post-heating (blue points) systems at each NMC composition with diferent delithiation states.

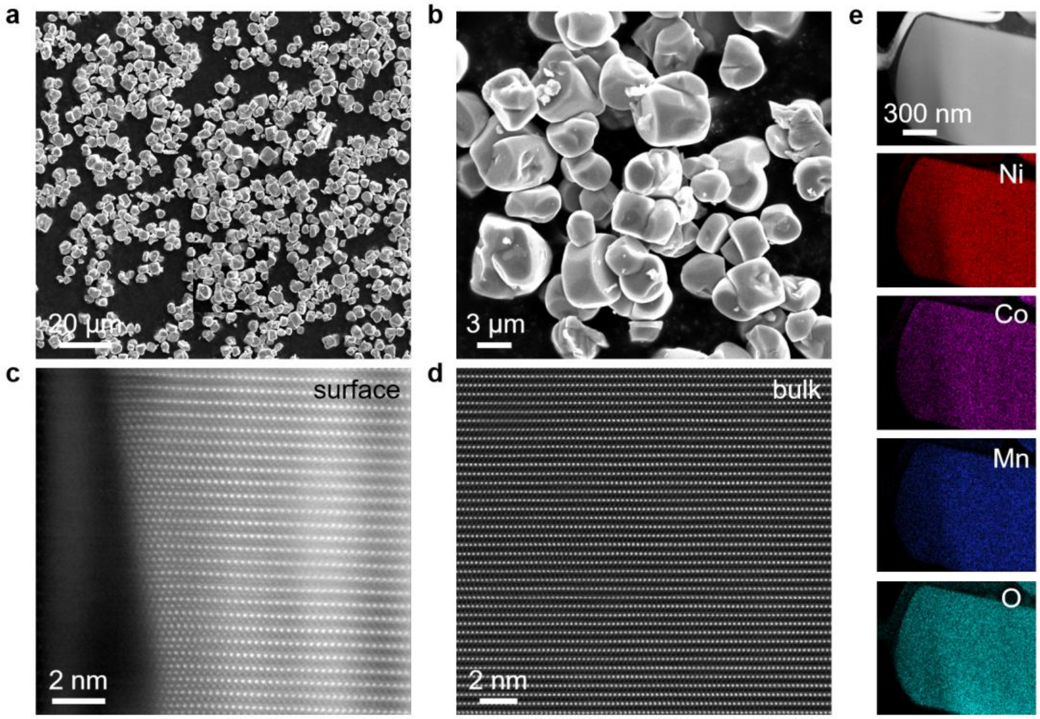

Supplementary Figure 2. a,**b**, SEM images of the single-crystalline NMC622. **c**,**d**, High-angle annual dark-field STEM images for the surface (**c**) and bulk (**d**) of the single-crystalline NMC622. **e**, STEM elemental mapping of the single-crystalline NMC622.

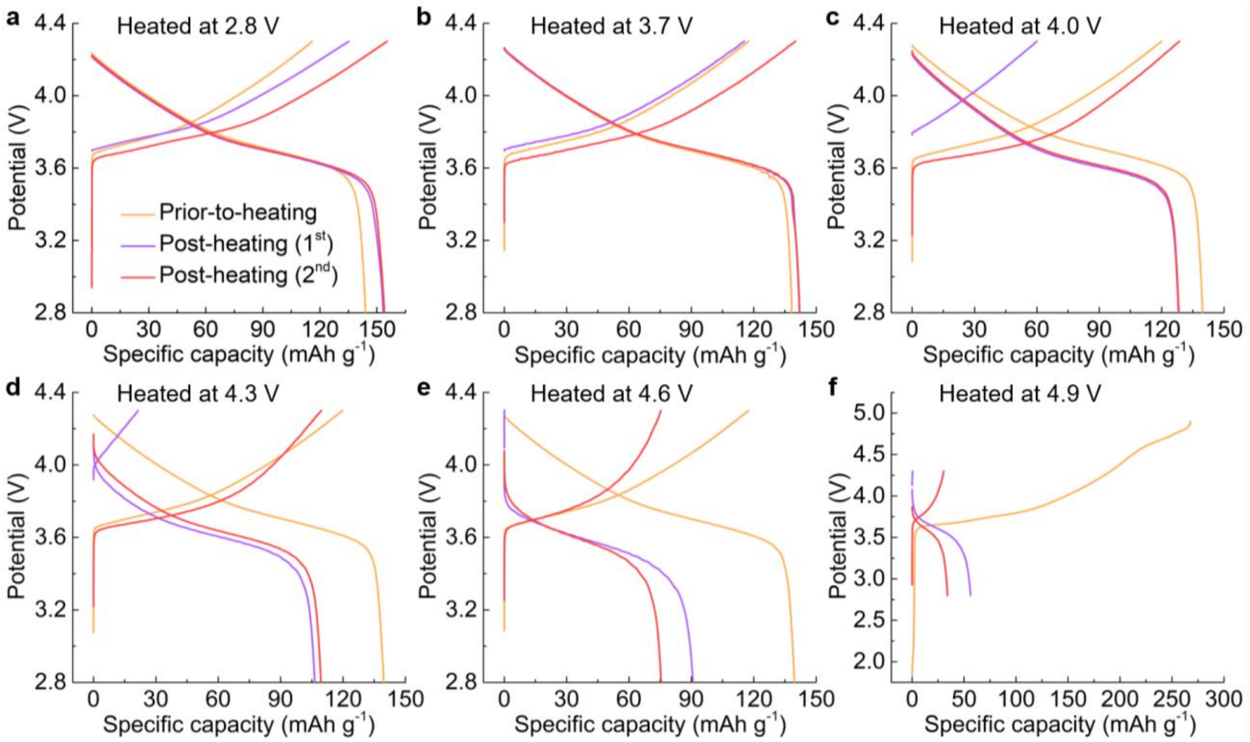

Supplementary Figure 3. a-f, Voltage profile of 2.8 V (**a**), 3.7 V (**b**), 4.0 V (**c**), 4.3 V (**d**), 4.6 V (**e**), 4.9 V (**f**) electrodes in the initial two cycles (after thermal treatment) in comparison with that of the pristine single-crystalline NMC622 electrode. The curve names in (**b**-**f**) are identical to (**a**). The voltage profile of 2.8, 3.7, 4.0, 4.3, 4.6 and 4.9 V electrodes correspond to Cell-8 (2.8 V), Cell-2 (3.7 V), Cell-2 (4.0 V), Cell-2 (4.3 V), Cell-4 (4.6 V), and Cell-4 (4.9 V) shown in Supplementary Table S2, respectively.

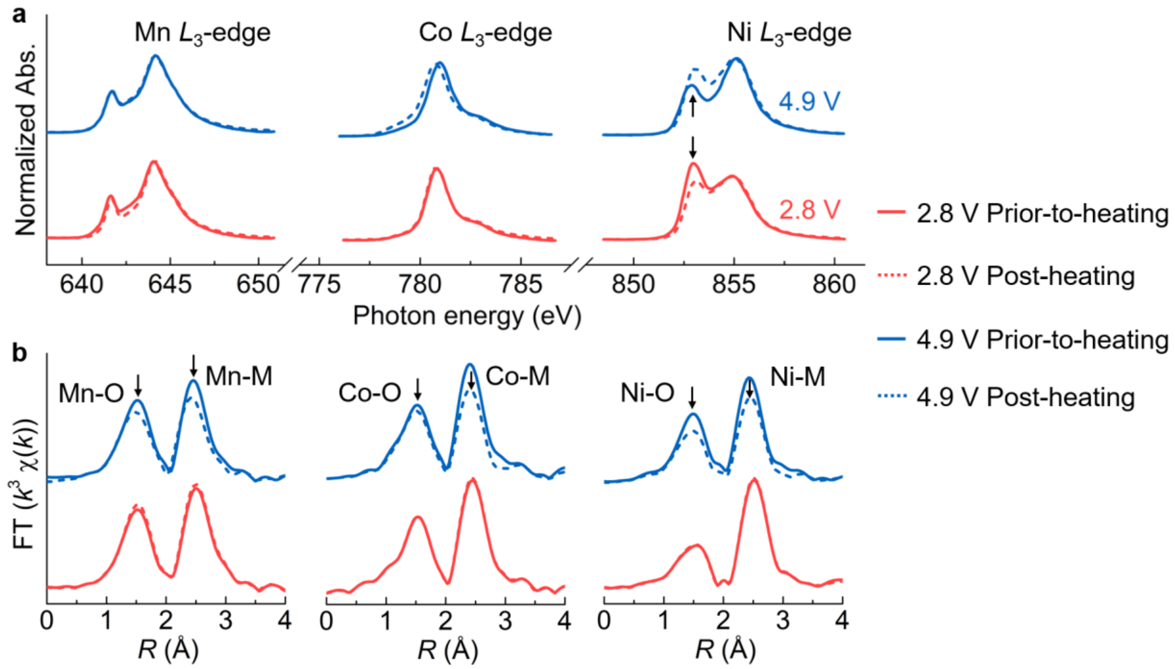

Supplementary Figure 4. a, Ni, Co, Mn L_3-edges spectra of the 2.8 V and 4.9 V electrodes before and after thermal treatment. **b**, FT-EXAFS spectra at Ni, Co, Mn K-edges for the 2.8 V and 4.9 V electrodes before and after thermal treatment.

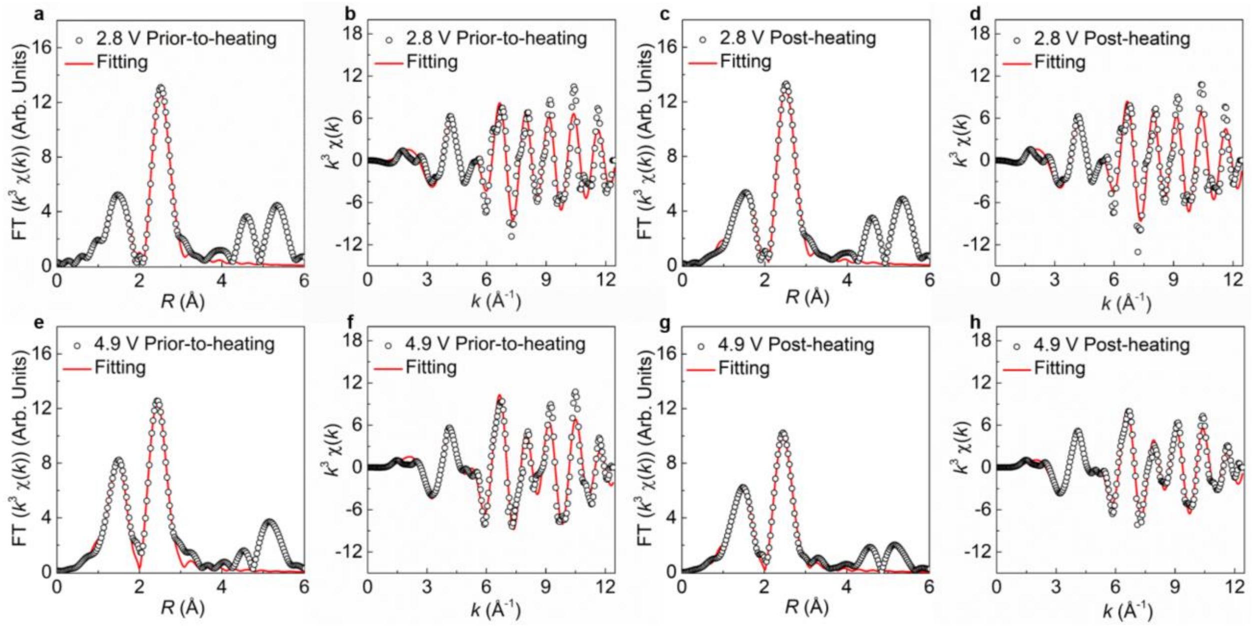

Supplementary Figure 5. a-h, EXAFS *R* space fitting and $k^3\chi(k)$ oscillation curves results of Ni K-edge for 2.8 V electrode before thermal treatment (**a**,**b**) and after thermal treatment (**c**,**d**), 4.9 V electrode before thermal treatment (**e**,**f**) and after thermal treatment (**g**,**h**).

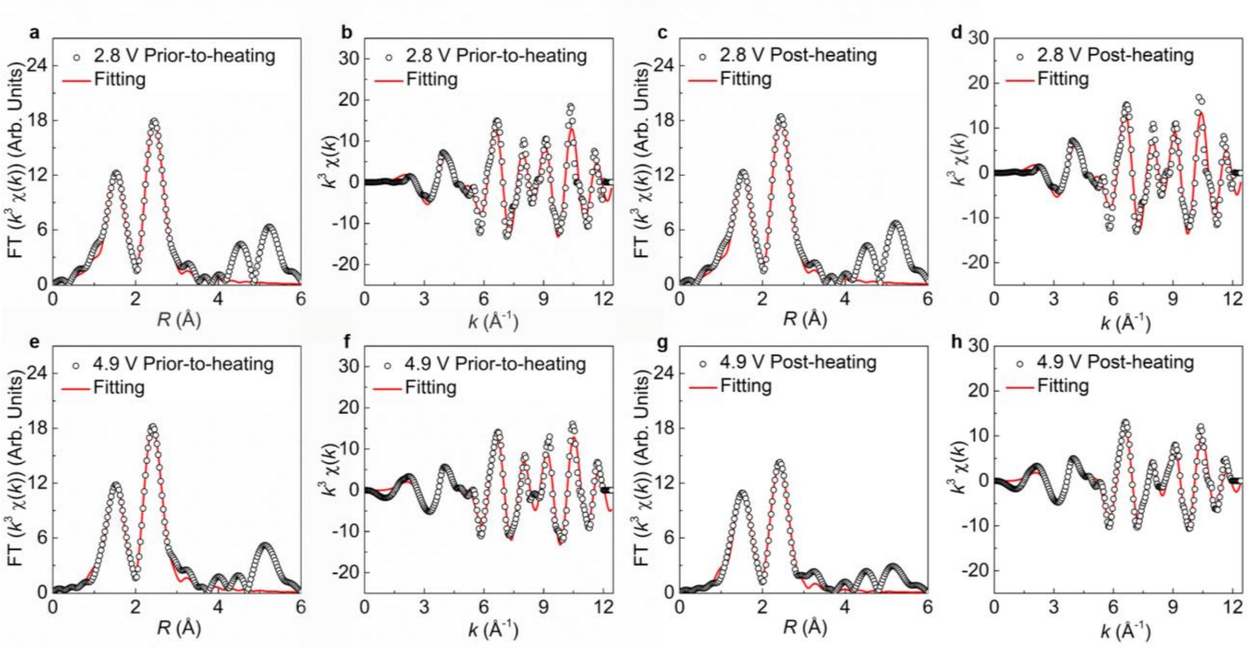

Supplementary Figure 6. a-h, EXAFS *R* space fitting and $k^3\chi(k)$ oscillation curves results of Co K-edge for 2.8 V electrode before thermal treatment (**a**,**b**) and after thermal treatment (**c**,**d**), 4.9 V electrode before thermal treatment (**e**,**f**) and after thermal treatment (**g**,**h**).

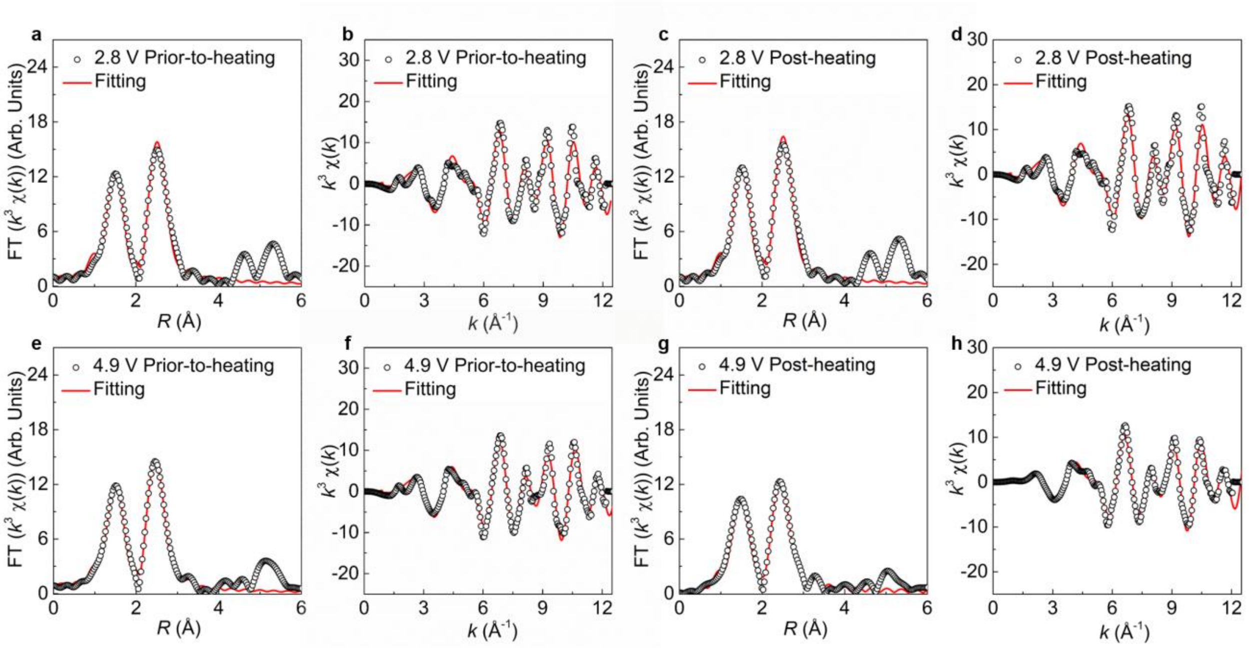

Supplementary Figure 7. a-h, EXAFS *R* space fitting and $k^3\chi(k)$ oscillation curves results of Mn K-edge for 2.8 V electrode before thermal treatment (**a**,**b**) and after thermal treatment (**c**,**d**), 4.9 V electrode before thermal treatment (**e**,**f**) and after thermal treatment (**g**,**h**).

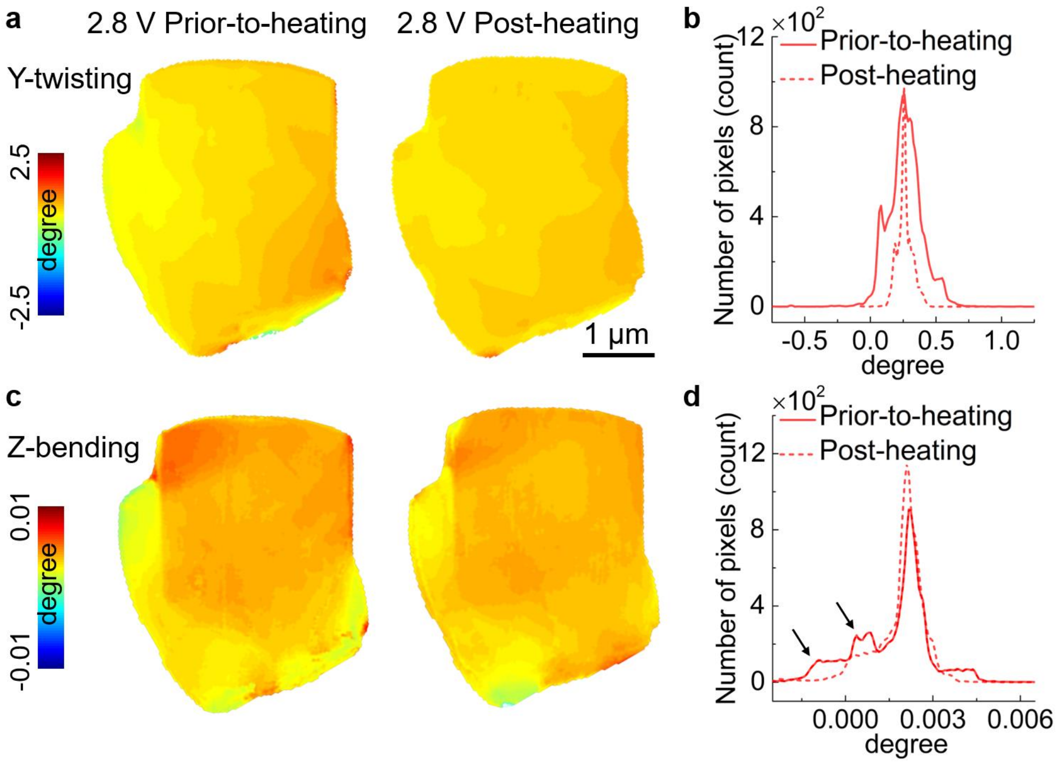

Supplementary Figure 8. a, The lattice strain map of Y-twisting for 2.8 V particle before and after thermal treatment. **b**, The relative probability distribution of the lattice strain displayed in (**a**). **c**, The lattice strain map of Z-bending for 2.8 V particle before and after thermal treatment. **d**, The relative probability distribution of the lattice strain displayed in (**c**).

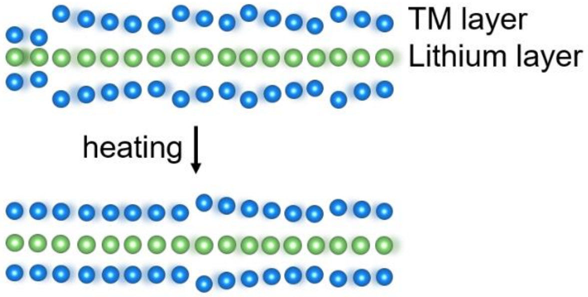

Supplementary Figure 9. Schematic illustration of the lattice arrangement in the 2.8 V particle during the thermal treatment.

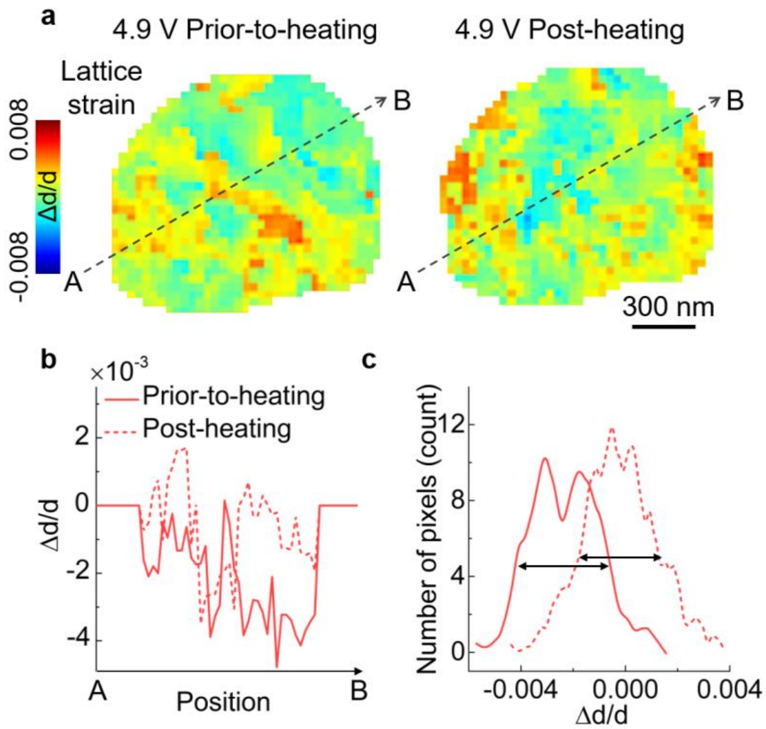

Supplementary Figure 10. **a**, The lattice strain map of d-spacing for 4.9 V particle before and after thermal treatment. **b**, The line profiles from points a to b as illustrated in (**a**). **c**, The relative probability distribution of the lattice strain displayed in (**a**). The curve name in (**c**) is identical to (**b**).

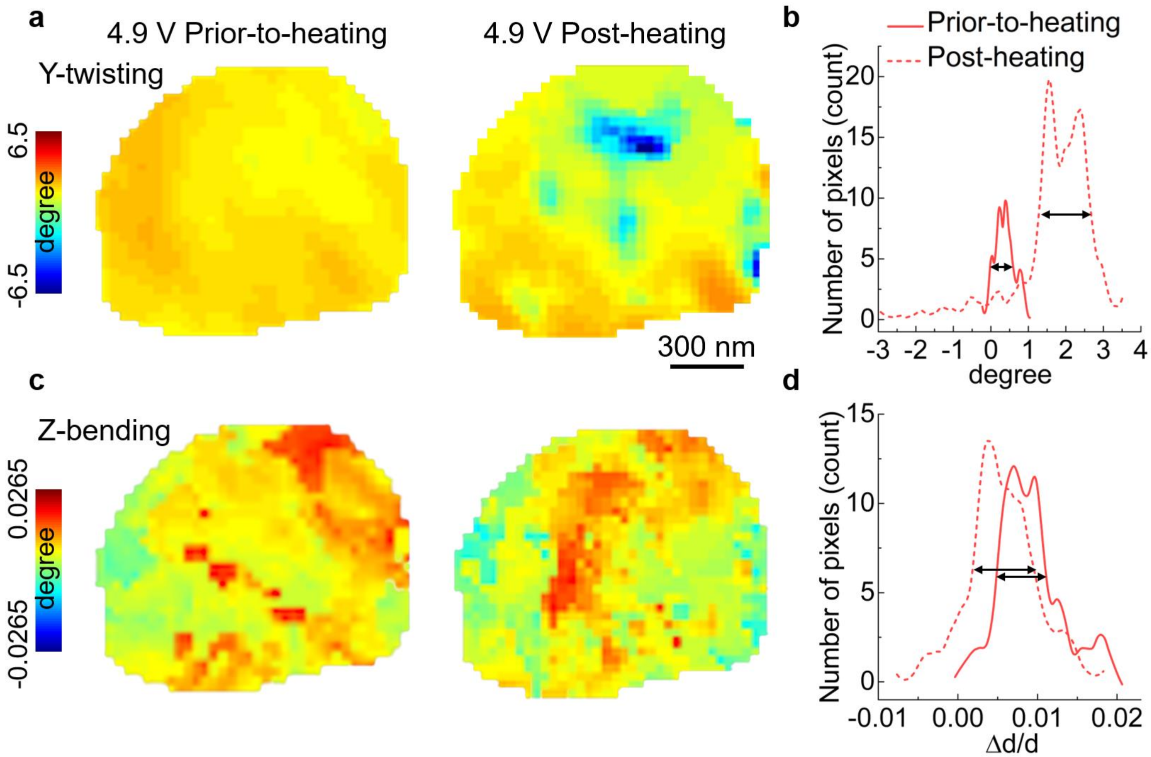

Supplementary Figure 11. a, The lattice strain map of Y-twisting for 4.9 V particle before and after thermal treatment. **b**, The relative probability distribution of the lattice strain displayed in (**a**). **c**, The lattice strain map of Z-bending for 4.9 V particle before and after thermal treatment. **d**, The relative probability distribution of the lattice strain displayed in (**c**). The curve name in (**d**) is identical to (**b**).

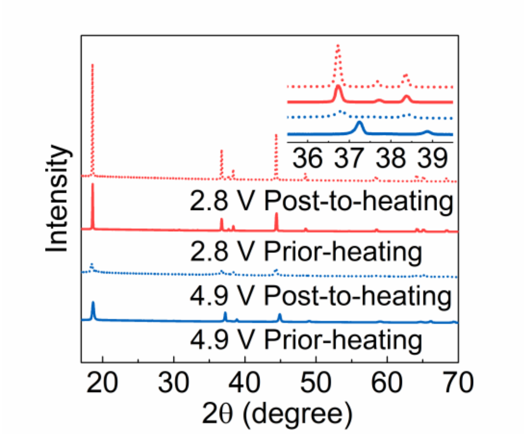

Supplementary Figure 12. XRD patterns of 2.8 V and 4.9 electrodes before and after thermal treatment. The inset is a magnified XRD patterns from 35.5^o to 39.5^o.

Supplementary Tables

Supplementary Table S1. Elemental composition of the single-crystalline NMC622 shown in molar ratio.

Sample	Li	Ni	Co	Mn
Single-crystalline NMC622	1.04	0.59	0.20	0.21

Supplementary Table S2. Capacity comparison of different electrodes before and after thermal treatment. All the post-heating capacities shown here are based on the voltage profile of different electrodes at the 2nd cycles.

Sample	Cell number	Prior-to-heating capacity (mAh g^{-1})	Post-heating capacity (mAh g^{-1})	Averaged capacity difference (mAh g^{-1})
2.8 V	Cell-1	141	148	10 =152-142
	Cell-2	139	159	
	Cell-3	142	148	
	Cell-4	144	158	
	Cell-5	146	148	
	Cell-6	139	152	
	Cell-7	139	149	
	Cell-8	144	154	
	Cell-9	142	149	
3.7 V	Cell-1	133	140	5 =140-135
	Cell-2	138	142	
	Cell-3	136	140	
	Cell-4	132	138	
	Cell-5	135	138	
4.0 V	Cell-1	142	132	-11 =130-141
	Cell-2	139	128	
	Cell-3	141	131	
	Cell-4	143	131	
4.3 V	Cell-1	144	110	-26 =116-142
	Cell-2	139	109	
	Cell-3	143	129	
	Cell-4	140	114	
4.6 V	Cell-1	136	85	-59 =79-138
	Cell-2	139	95	
	Cell-3	139	61	
	Cell-4	139	75	
4.9 V	Cell-1	140.1	51	-103 =37-140
	Cell-2		29	
	Cell-3		34	
	Cell-4		33	

Supplementary Table S3. Capacity comparison of the electrodes before and after reassembly.

Sample	Cell number	Prior-reassembly (mAh g^{-1})	Post-reassembly (mAh g^{-1})	Averaged capacity difference (mAh g^{-1})
Reassembly	Cell-1	138	133	1<br>=140-139
	Cell-2	138	145	
	Cell-3	140	143	

Supplementary Table S4. EXAFS fitting results for the structural parameters around Ni atoms in 2.8 V and 4.9 V electrodes before and after thermal treatment.

Sample	Path	N	R(Å)	σ^2 (10^{-3}Å)	ΔE_0 (eV)
2.8 V Prior-to-heating	Ni-O	6.0±0.1	1.94±0.02	9.6±0.6	-1.5±0.5
	Ni-M	6.0±0.2	2.86±0.01	4.4±0.5	-0.9±0.4
2.8 V Post-heating	Ni-O	6.0±0.1	1.94±0.02	9.1±0.6	-1.1±0.6
	Ni-M	6.0±0.2	2.86±0.01	4.2±0.4	-1.1±0.7
4.9 V Prior-to-heating	Ni-O	5.0±0.2	1.89±0.03	5.2±0.6	-3.1±0.5
	Ni-M	5.7±0.1	2.81±0.01	5.5±0.5	-5.0±0.4
4.9 V Post-heating	Ni-O	4.5±0.2	1.90±0.02	5.8±0.6	-4.9±0.8
	Ni-M	4.6±0.1	2.84±0.01	5.3±0.8	-5.9±0.8

N is the coordination number; R is interatomic distance (the bond length between Co central atoms and surrounding coordination atoms; σ^2 is Debye-Waller factor (represents the thermal and static disorder in absorber-scatterer distances); ΔE_0 is edge-energy shift (the difference between the zero kinetic energy value of the sample and that of the theoretical model).

Supplementary Table S5. EXAFS fitting results for the structural parameters around Co atoms in 2.8 V and 4.9 V electrodes before and after thermal treatment.

Sample	Path	N	R(Å)	σ^2 (10^{-3}Å)	ΔE_0 (eV)
2.8 V Prior-to-heating	Co-O	6.0±0.2	1.92±0.02	2.2±0.6	-3.0±0.5
	Co-M	6.0±0.2	2.84±0.03	2.5±0.5	-7.7±0.4
2.8 V Post-heating	Co-O	6.0±0.2	1.92±0.02	2.1±0.6	-3.2±0.6
	Co-M	6.0±0.1	2.84±0.01	2.3±0.4	-7.9±0.7
4.9 V Prior-to-heating	Co-O	6.0±0.2	1.91±0.03	2.5±0.6	-1.6±0.5
	Co-M	6.0±0.2	2.81±0.02	2.3±0.5	-7.3±0.4
4.9 V Post-heating	Co-O	5.6±0.2	1.91±0.02	2.3±0.6	-1.9±0.5
	Co-M	5.6±0.1	2.84±0.01	4.3±0.5	-9.5±0.4

Supplementary Table S6. EXAFS fitting results for the structural parameters around Mn atoms in 2.8 V and 4.9 V electrodes before and after thermal treatment.

Sample	Path	*N*	*R*(Å)	σ^2 (10^{-3}Å)	ΔE_0 (eV)
2.8 V Prior-to-heating	Mn-O	6.0±0.2	1.91±0.02	1.8±0.6	3.4±0.5
	Mn-M	5.0±0.2	2.88±0.01	1.2±0.5	2.0±0.4
2.8 V Post-heating	Mn-O	6.0±0.2	1.91±0.02	1.4±0.6	3.1±0.6
	Mn-M	5.0±0.1	2.88±0.01	0.9±0.4	1.8±0.7
4.9 V Prior-to-heating	Mn-O	6.0±0.2	1.90±0.03	2.1±0.6	3.2±0.5
	Mn-M	5.0±0.2	2.85±0.02	1.1±0.5	1.4±0.4
4.9 V Post-heating	Mn-O	5.8±0.2	1.90±0.02	2.0±0.6	-5.4±0.5
	Mn-M	4.0±0.1	2.87±0.01	1.0±0.5	-7.6±0.4
